# Genistein Promotes Endothelial Colony-Forming Cell (ECFC) Bioactivities and Cardiac Regeneration in Myocardial Infarction

**DOI:** 10.1371/journal.pone.0096155

**Published:** 2014-05-15

**Authors:** Sang Hun Lee, Jun Hee Lee, Takayuki Asahara, Yong Sook Kim, Hae Chang Jeong, Youngkeun Ahn, Jin Sup Jung, Sang-Mo Kwon

**Affiliations:** 1 Medical Science Research Institute Soonchunhyang University Seoul Hospital Seoul, Korea; 2 Laboratory for Vascular Medicine & Stem Cell Biology, Medical Research Institute, Department of Physiology, School of Medicine, Pusan National University Yangsan, Korea; 3 Immunoregulatory Therapeutics Group in Brain Busan 21 Project, Department of Physiology, School of Medicine, Pusan National University Yangsan, Korea; 4 Stem Cell Translational Research, Institute of Biomedical Research and Innovation/RIKEN Center for Developmental Biology, Kobe, Japan; 5 Heart Research Center, Chonnam National University Hospital, Gwangju, Korea; 6 Department of Cardiology, Chonnam National University Hospital, Gwangju, Korea; 7 Department of Physiology, School of Medicine, Pusan National University, Yangsan, Gyeongnam, Korea; 8 Department of Medical Bioscience, Soonchunhyang University Asan, Asan, Korea; 9 Department of Regenerative Medicine Science, Tokai University School of Medicine, Isehara, Japan; University of Central Florida, United States of America

## Abstract

Although stem cell-mediated treatment of ischemic diseases offers significant therapeutic promise, the limitation in the therapeutic efficacy of transplanted stem cells *in vivo* because of poor engraftment remains a challenge. Several strategies aimed at improving survival and engraftment of stem cells in the ischemic myocardium have been developed, such as cell transplantation in combination with growth factor delivery, genetic modification of stem cells, and/or cell therapy using scaffolds. To improve therapeutic efficacy, we investigated the effects of genistein on the engraftment of transplanted ECFCs in an acute myocardial ischemia model. *Results*: We found that genistein treatment enhanced ECFCs' migration and proliferation, which was accompanied by increases in the expression of ILK, α-parvin, F-actin, and phospholylation of ERK 1/2 signaling. Transplantation of genistein-stimulates ECFCs (GS-ECFCs) into myocardial ischemic sites *in vivo* induced cellular proliferation and secretion of angiogenic cytokines at the ischemic sites and thereby enhanced neovascularization and decreased myocardial fibrosis as well as improved cardiac function, as shown by echocardiography. Taken together, these data suggest that pretreatment of ECFCs with genistein prior to transplantation can improve the regenerative potential in ischemic tissues, providing a novel strategy in adult stem cell therapy for ischemic diseases.

## Introduction

Several studies have recently reported promising results by modifying and enhancing stem cell-mediated ischemic myocardial repair and regeneration [1]–[6]. Increasing evidence from experimental ischemic animal models suggests that endothelial progenitor cells (EPCs) participate in the process of neovascularization and tissue repair, leading to enhanced recovery of the ischemic myocardium [7]–[10]. EPCs are also known as endothelial colony-forming cells (ECFCs) or late EPCs. Clinical trials involving ECFC transplantation for ischemic myocardium have confirmed this possibility [11]–[13]. However, the adverse effects of ischemic tissue on the survival and function of the transplanted ECFC during angio/vasculogenesis and tissue repair is still a poses a challenge and research on the means to enhance stem cell survival and function is limited. Thus we propose new method of augmenting neovascularization by overcoming the poor engraftment of ECFCs into ischemic tissue and enhancing its ECFCs survival.

Endothelial progenitor cells are thought to promote neovasculogenesis by 2 separate mechanisms. First, bone marrow-derived EPCs have been shown to incorporate themselves into newly formed vessels, crossing from the circulation into the interstitium via a process that is similar to neutrophil adhesion and endothelial transmigration [14]–[15]. This mechanism has been extensively studied, with most investigations focused on providing EPCs as the building blocks of new vessels. However, translation of these experimental observations to human clinical trials has been plagued by the large number of cells required to demonstrate a clinical benefit. Second, in addition to the ability of EPCs to form new vessels, they also produce proangiogenic cytokines that induce the growth of new blood vessels by promoting the migration and proliferation of local endothelial cells [16]–[18]. Several groups have demonstrated a therapeutic benefit by administering these proangiogenic factors directly into the myocardium [19]. The known factors include, but are not limited to, estrogen (E_2_), vascular endothelial growth factor (VEGF), and stromal cell-derived factor-1α (SDF). Each of these factors plays a specific role in the angiogenic cascade. E_2_ and VEGF promote endothelial cell proliferation and subsequent angiogenesis [20], whereas SDF functions as a chemotactic factor for the recruitment and activation of additional EPCs.

Genistein, an isoflavone derived from soybeans, has a weak affinity for estrogen receptor-α, which is present in reproductive organs; In contrast, the affinity of genisteinfor estrogen receptor-β, which is present in the vasculature, is similar to that of estrogen. Therefore, it can be administered to both sexes [21]. Genistein has been shown to protect against myocardial ischemia-reperfusion injury in a rat model when administered acutely [22]. Genistein also improves endothelium-dependent vasodilation in ovariectomized rats after 4 weeks of therapy [23] and in postmenopausal women after 6 months of therapy [24]. These reports suggest that the therapeutic applications of genistein for vascular repair are similar to those of estrogen. Here, we investigate the role of genistein (a plant-derived estrogen) on the bioactivity of endothelial colony forming cells (ECFCs) to define its potential therapeutic impact on myocardial regeneration after infarction, which may provide a new method for improved engraftment of ECFCs into ischemic tissues by augmenting neovascularization and enhancing ECFC survival.

## Results

### Effect of genistein on ECFC migration and proliferation

**Figure 1:**
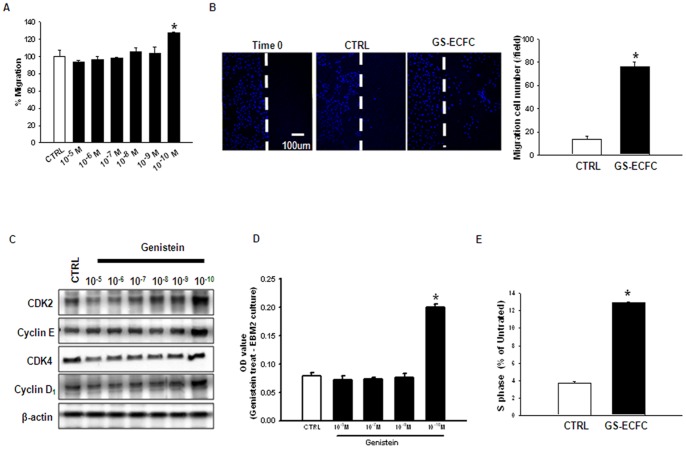
Effect of genistein on ECFC migration and proliferation. (A) ECFCs were incubated for 12 h with various concentrations of genistein (10^−10^–10^−5^ M) and then stained with 5 µM Calcein AM. Fluorescence was quantified with a plate reader. (B) The *in vitro* ECFC wound-healing motility assay was performed in the absence and presence of genistein. Ten fields per plate were examined. (Scale bar: 100 µm). (C) ECFCs were incubated for 12 h with various concentrations of genistein, and CDK 2, cyclin E, CDK 4, and cyclin D_1_ were assessed by western blotting. (D) ECFCs were incubated for 12 h with various concentrations of genistein, and assessed by MTT. (E) ECFCs were treated with genistein and then washed with PBS, fixed, stained, and analyzed by flow cytometry. Gates were manually configured to determine the percentage of cells in S phase based on the DNA content (*n* = 5). *P<0.05 vs. CTRL (indicates control genistein untreated ECFCs).

The migration and proliferation of ECFCs incubated with various concentrations (10^10^–10^−5^ M) of genistein were examined. Figure 1A shows that genistein at 10^−10^ M significantly increased ECFC migration. Increased cell migration was observed after 12 h incubation with genistein (10^−10^ M) (Fig. 1B). Genistein-induced ECFC proliferation was also studied with ECFCs incubated with various concentrations of genistein (10^10^–10^−5^ M). Genistein at 10^−10^ M significantly increased the cell cycle regulatory protein expression levels (Fig. 1C). The effect of genistein on cell proliferation was examined by treating ECFCs with genistein with various doses for 12 h. As shown in Figure 1D, Genistein at 10^−10^ M significantly increased the cell proliferation. Genistein exposure (10^−10^ M) for 12 h resulted in a significant increase in the percentage of cells in the S-phase (Fig. 1E). These results suggest that genistein may enhance migration and promote proliferation in ECFCs at low concentrations, which were reduced at high concentrations.

### Genistein-stimulated homing of ECFCs to ischemic tissue is accompanied by enhanced expression of ILK, α-parvin, F-actin

**Figure 2:**
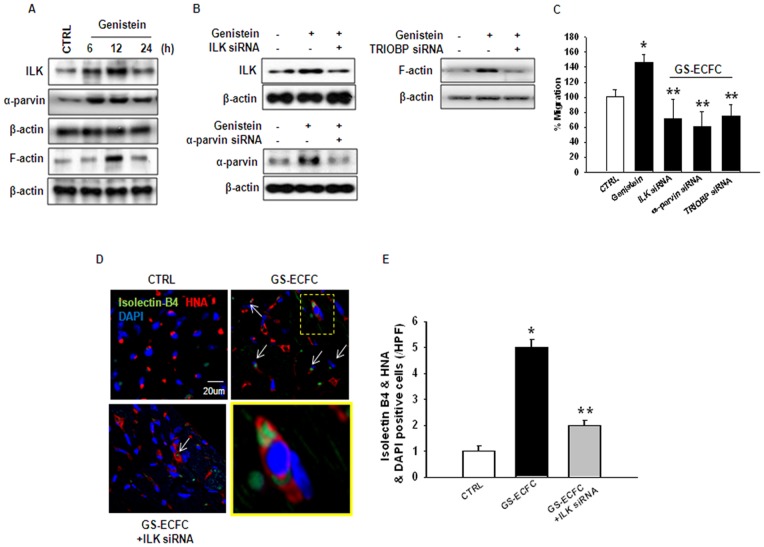
ILK, α-parvin and F-actin mediated genistein-induced ECFC migration. (A) ECFCs were treated with genistein for 0–24 h, and ILK, α-parvin and F-actin was detected by western blotting. (B) ECFCs were transfected with ILK, α-parvin, and TRIOBP (F-actin) small interfering RNA (siRNA) (ILK, α-parvin, and TRIOBP-specific siRNA; 200 pmol) for 24 h before genistein treatment and staining with Calcein AM. Fluorescence in the analytical zone was quantified with a plate reader. *P<0.05 vs. CTRL (indicates control genistein untreated ECFCs), **P<0.05 vs. genistein. (C) ECFCs were transfected with ILK siRNA (ILK-specific siRNA; 200 pmol) for 24 h before genistein (10^−10^ M) treatment, and the cells were injected into the tail veins of mice 30 min after left anterior descending (LAD) artery ligation. Staining of ECs with isolectin B4 (green) showed human nuclei antibody (HNA) (red)-positive cell incorporation into the border zone of left ventricular (LV) infarct at 3 days after myocardial infarction (MI) (Scale bar: 20 µm). **Inset** in higher magnification of the **yellow-boxed area**. **Arrows** indicate of isolectin B4+HNA+DAPI+cells. (D) The bar graph shows quantitative analysis of the number of HNA+cells associated with isolectin B4+vasculature (n = 5). HPF indicates high-power field. *P<0.05 vs. CTRL (indicates control genistein untreated ECFC), **P<0.05 vs. genistein stimulate-ECFC (GS-ECFC).

To determine whether genistein plays a role in regulating ILK, α-parvin and F-actin expression, ILK, α-parvin and F-actin were analyzed by western blot. Genistein increased ILK, α-parvin and F-actin expression in cell lysates (Fig. 2A). Also, ILK, α-parvin and TRIOBP-specific siRNA reduced the genistein-induced increased in ILK, α-parvin and F-actin levels (Fig. 2B). To further elucidate the involvement of ILK, α-parvin and F-actin in the genistein-induced cell migration, ECFCs were transfected with ILK, α-parvin and TRIOBP-specific siRNA prior to genistein treatment. ILK, α-parvin and TRIOBP-specific siRNA reduced the genistein-induced increase of cell migration (Fig. 2C). These *in vitro* results raised the possibility that genistein enhances the homing of ECFCs to the injured myocardium, favoring recovery of an infarcted heart. In comparison with the CTRL (control untreated ECFSs) injected mice, the injection of the genistein stimulated-ECFC (GS-ECFC) group resulted in a 4-fold increase in the number of isolectin B4/HNA/DAPI-positive ECFCs, whereas ILK-specific siRNA transfection GS-ECFC (ILK siRNA+GS-ECFC) blocked the homing (Fig. 2D and E). These results support the suggestion that genistein increases ECFC motility via the ILK pathway.

### Involvement of the ERK1/2 pathway in genistein-induced ECFC proliferation and enhances survival of ECFCs in myocardial ischemic tissues

**Figure 3:**
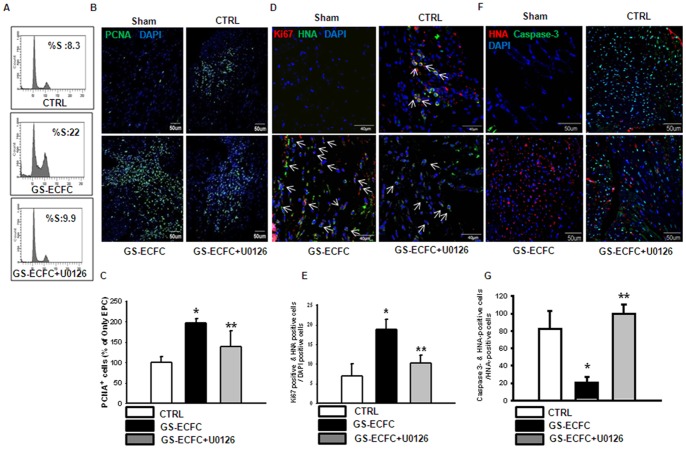
ERK1/2-mediated genistein-induced ECFC proliferation and survival in the border zone of the left ventricular (LV) infarct at 3 days after myocardial infarction (MI). (A) ECFCs were pretreated with U0126 (ERK1/2 inhibitor, 10^−6^ M) for 30 min prior to 12 h of genistein treatment and then washed with PBS, fixed, stained, and analyzed by flow cytometry. Gates were manually configured to determine the percentage of cells in S phase based on DNA content (n = 5). *P<0.05 vs. CTRL (indicates control genistein untreated ECFC), **P<0.05 vs. genistein stimulate-ECFC (GS-ECFC). ECFCs were pretreated with U0126 for 30 min prior to a 12 h genistein (10^−10^ M) treatment, and the cells were transplanted into the ischemic region. (B) Proliferating cell nuclear antigen (PCNA) staining to detect ECFC proliferation. PCNA+ cell zone, **yellow-boxed area.** (Scale bar: 100 µm). (C) Quantification of PCNA-positive cells at 3 d after MI. (D) Co-immunofluorescent staining to detect proliferation (Ki67 [proliferation marker, red] and of hECFCs [human nuclear antigen (HNA)-positive cells, green] and DAPI [blue] for nuclear staining). **Arrows** indicate Ki67+ HNA+ DAPI+ cells. (E) Quantitative analysis of Ki67/HNA/DAPI triple-positive cells at 3 days after MI. (F) Coimmunofluorescent staining to detect apoptosis (caspase-3, apoptosis marker, green) and of hEPCs (HNA-positive cells, red) and DAPI (blue) by nuclear staining. **Arrows** indicate caspase-3+ HNA+ DAPI+ cells. (Scale bar: 20 µm). (G) Quantitative analysis of caspase-3/HNA/DAPI triple-positive cells at 3 days after MI. (n = 6) *P<0.05 vs. CTRL (indicates control genistein untreated ECFC), **P<0.05 vs. genistein stimulate-ECFC (GS-ECFC).

To assess the involvement of ERK1/2 in genistein-induced ECFC proliferation, the genistein-induced increase in the percentage of cell population in the S phase was significantly blocked by U 0126 (ERK1/2 inhibitor, 10^−6^ M) (Fig. 3A). These results support the suggestion that genistein increases ECFC proliferation via the ERK1/2 pathway. In the previous *in vitro* experiments, we demonstrated that culturing ECFCs in genistein-containing medium activated the ERK1/2 signaling pathway and increased their proliferative potential. We hypothesized that genistein pretreatment of ECFCs would be beneficial for repairing the damaged tissue in the myocardial ischemia injury model by preparing the cells with better survival rate to the site of ischemic injury. As shown in Fig. 3B and C, more PCNA-positive cells were found when genistein stimulate-ECFCs (GS-ECFCs) were transplanted than when CTRL (control untreated ECFCs) were transplanted. Indeed, HNA and proliferating cell marker (Ki67)-double positive cells at 3 days were more abundant in the case of the genistein stimulate-ECFCs (GS-ECFCs) than in that of CTRL (control untreated ECFCs) (Fig. 3D and E). Furthermore, IF staining of caspase-3 and HNA in ischemic muscle 3 days after transplantation showed that apoptotic ECFCs were significantly less after grafting of genistein stimulate-ECFCs (GP-ECFCs) than after grafting CTRL (control genistein untreated ECFCs). However, ERK1/2 inhibitor (U0126, 10^−6^ M)-pretreatment GS-ECFCs showed poor proliferation and survival in ischemic heart tissue (Fig. 3F and G).

### Genistein stimulates ECFCs to secrete paracrine cytokines leading to cell neovascularization

**Figure 4:**
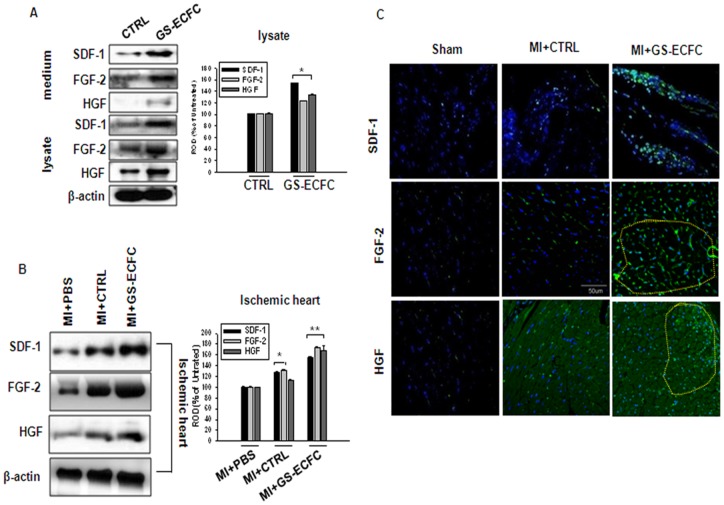
Enhanced secretion of angiogenic growth factors in the ischemic border zone following transplantation of GS-ECFCs. (A) ECFCs were cultured in serum-free medium for 24 h and then treated with genistein (10^−10^ M) for 12 h. SDF-1, HGF, and FGF-2 were determined by western blotting. (B) Western blot analyses of SDF-1, HGF, and FGF-2 at 3 day after genistein stimulate-ECFC (GS-ECFC) or CTRL (indicates control genistein untreated ECFC) were transplanted. The lower parts (A, B) depict the mean ± SE of 4 independent experiments for each condition, as determined from densitometry relative to β-actin. P<0.05 vs. CTRL (indicates control genistein untreated ECFC), **P<0.05 vs. genistein stimulate-ECFC (GS-ECFC). (C) IF staining for SDF-1, HGF, and FGF-2 in ischemic heart tissue at 3 days after ECFCs were grafted as genistein stimulate-ECFC (GS-ECFC) or CTRL (indicates control genistein untreated ECFC) (Cytokine expression zone, **yellow-boxed area**) (Scale bar: 50 µm) (n = 5).

Transplantation of genistein stimulate-ECFCs (GS-ECFCs) into the ischemic tissues enhanced paracrine secretion of angiogenic growth factors. Genistein induced the increase in human angiogenic growth factor (SDF-1, HGF, and FGF-2) secretion and expression in ECFCs (Fig. 4A). Western blot analysis showed that human angiogenic growth factor expression in tissues was more extensive in genistein stimulate-ECFCs (GS-ECFCs) transplantation relative to CTRL (control genistein untreated ECFCs) (Fig. 4B). IF staining for the human angiogenic growth factors SDF-1, HGF, and FGF-2 indicated that secretion from transplanted genistein stimulate-ECFCs (GS-ECFCs) began at 3 days after transplantation, whereas most transplanted CTRL (control genistein untreated ECFCs) did not secrete angiogenic growth factors until after 3 days (Fig. 4C).

**Figure 5:**
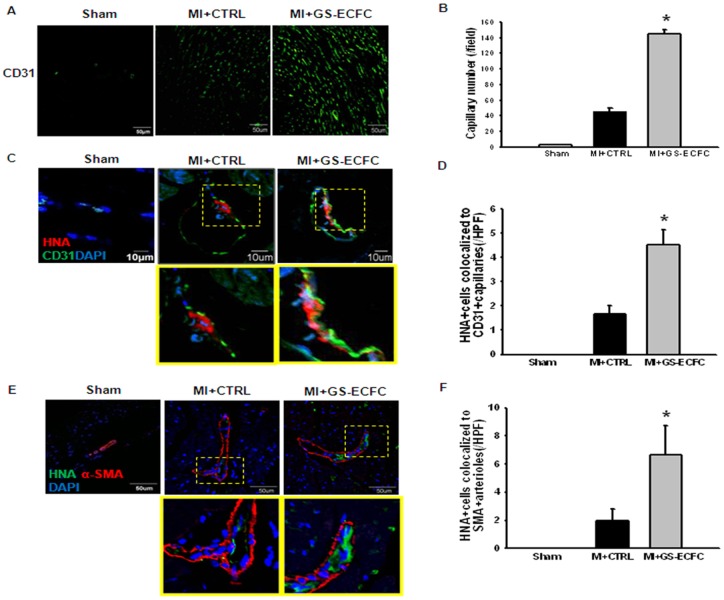
GS-ECFC-mediated neovascularization in border zone of LV infarct at 28 days post-MI. (A) IF staining for CD 31 (green) in ischemic heart tissue at 28 days after ECFCs were grafted as genistein stimulate-ECFC (GS-ECFC) or CTRL (control untreated ECFC). (Scale bar: 50 µm). (B) The bar graph shows the quantification of CD31^+^ capillary density. (C) Engraftment of ECFC (HNA+, red fluorescence) into vascular structures (CD31 staining for capillaries green fluorescence) is seen as yellow structures. **Insert** is higher magnification of the **yellow-boxed area**. (D) The bar graph shows the quantification of HNA^+^ cells associated with CD31+ vasculature. (E) Engraftment of ECFC (HNA+, green fluorescence) into arteriole structures (α-SMA staining for arterioles red fluorescence) is seen as yellow structures. **Insert** is higher magnification of the **yellow-boxed area**. (F) The bar graph shows the quantification of HNA^+^ cells associated with α-SMA + arterioles. HPF indicates high-power field. (n = 6) *P<0.05 vs. CTRL (indicates control genistein untreated ECFC).

Transplantation of genistein stimulate-ECFCs (GS-ECFCs) promoted angiogenesis in myocardial ischemic tissues. IF staining for CD31 and quantification of capillary density revealed that transplantation of genistein stimulate-ECFCs (GS-ECFCs) significantly enhanced the capillary formation compared to transplantation of CTRL (control genistein untreated ECFCs) (Fig. 5A–D). Similarly, IF staining for α-SMA (Fig. 5E and F) showed that arteriole formation was enhanced by transplanting genistein stimulate-ECFCs (GS-ECFCs). Ischemic tissues transplanted with genistein stimulate-ECFCs (GS-ECFCs) contained a larger number of transplanted EPCs (HNA-positive cells) than those with CTRL (control genistein untreated ECFCs).

### Genistein prestimulation enhanced ECFC therapeutic potential

**Figure 6:**
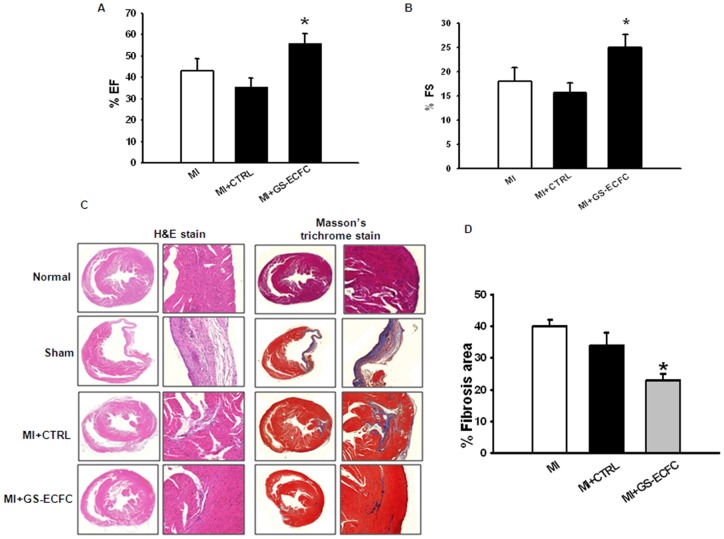
Enhancement of functional recovery by transplantation of GS-ECFC. M-mode echocardiographic tracing at 28 days after myocardial infarction (MI) in the CTRL (control untreated ECFC) and primed with genistein stimulate-ECFC (GS-ECFC) groups. Analysis of left ventricular (LV) diameter in (A) %EF and (B) %FS. %EF, percent of ejection fraction; and %FS, percent of functional shortening. (C) H&E and Trichrome-stained heart sections (28 days after MI). (D) The bar graph shows quantitative analysis of the fibrosis area at 28 days after MI (n = 9). *P<0.05 vs. CTRL (indicates control genistein untreated ECFC).

To determine if genistein promotes ECFC repair capability, LV function was assessed by echocardiography as described previously (Kawamoto et al., 2001; Kawamoto et al., 2003; Iwasaki et al., 2006) at 28 days post-MI. LVESD and LVEDD dimensions were measured by M-mode tracing, and the percent ejection fraction (%EF) and %FS were calculated. Mice that received 2.5×10^4^, ECFCs (low density cell number) showed worse function with reduced LVESD and insignificantly decreased %EF and %FS at 28 days post-MI compared to those with induced MI. Interestingly, genistein pretreatment robustly enhanced ECFC-induced improvement in LV function with a significant increase in %EF and %FS (Fig. 6A and B). To assess the influence of genistein stimulate-ECFCs (GS-ECFCs)-mediated LV remodeling effects, the percent fibrosis area was assessed at 28 days after MI. As expected, genistein stimulate-ECFCs (GS-ECFCs) significantly attenuated percent fibrosis (Fig. 6C, D). The current protocol of combining low-density with genistein culture successfully expanded a great amount of efficient ECFCs in a short time, and therefore can be applied in clinical practices.

## Discussion

Coronary artery disease is a significant cause of morbidity and mortality. Several repair mechanisms are now thought to involve circulating ECFCs mobilized from the bone marrow to sites of ischemic injury. A growing body of evidence in experimental cardiac injury models suggests that stem cell treatment or transplantation remodels and regenerates injured tissue improves function and protects from further insults. Indeed, these encouraging results have led to phase I and II clinical trials involving ECFC therapy for a variety of diseases [28]–[30]. However, stem cells transplanted into the ischemic myocardium are susceptible to adverse tissue microenvironments such as reduced oxygen supply and free radical damage, thereby compromising their full therapeutic benefits. Previous studies have shown that genistein, a phytoestrogen, protects against myocardial ischemia-reperfusion injury in a rat model [31]. However, whether genistein-mediated improvement of LV function and remodeling in injured myocardium can also modulate survival and function of transplanted ECFCs is unknown. Therefore, we tested if genistein could modulate ECFC biology leading to enhanced survival and function and therefore attenuate MI-induced LV dysfunction and remodeling after transplantation into the ischemic myocardium.

In most cases, genistein significantly inhibits cellular growth at high concentrations, but stimulates the growth at low concentrations [32]–[34]. In this study, genistein at 10^−10^ M significantly increased the migrating cell numbers. Additionally, genistein at 10^−10^ M increased the percentage of cells in the S phase during 12 h incubation (Figure 1). These findings strongly suggest that a low concentration of genistein plays a pivotal role in enhancing ECFC bioactivity. As a general rule, the primary action of genistein is mediated by ERs. Therefore, we initially examined the correlation between genistein and ERs in ECFC. Genistein treatment of ECFC increased the estrogen receptor (ER) expression (Figure S2). Cell migration is a complex process that is critically involved in embryonic development and many physiological and pathological processes including injury repair, inflammation, and metastasis. The integrin-linked kinase-PINCH-parvin (IPP) complex is linked to actin cytoskeletal activities via parvin as well as proteins bound to PINCH1 and ILK [35]. The actin cytoskeleton provides a structural framework around which cell shape and polarity are defined. Its dynamic properties provide the driving force for cells to move and divide. For instance, when integrin-linked kinase (ILK), a crucial binding partner of an integrin cytoplasmic domain, is ablated in fibroblasts, cell shape spreading, F-actin aggregation, focal adhesion formation and proliferative rates are impaired [36]. Further downstream of integrins, members of the Rho family of small guanosine triphosphatases (GTPases) have emerged as key regulators of the actin cytoskeleton, and furthermore, through their interaction with multiple target proteins, these Rho GTPases ensure the co-ordinated control of other cellular activities such as gene transcription and adhesion [37]. It has been suggested that the strength of focal adhesions influences cell motility [38]. In this study, we found that genistein promoted ECFC migration via an increase in ILK, α-parvin, and F-actin expression. We also demonstrated that genistein-induced stimulation of cell migration was blocked by ILK, α-parvin, and F-actin-specific siRNA transfection. These results suggest that expression of the ILK, α-parvin, and F-actin plays an important role in genistein-induced ECFC migration. In a nude mouse myocardium ischemic model, animals treated with genistein stimulate-ECFCs (GS-ECFCs) showed more motile activity that was composed of injected ECFCs than animals treated with CTRL (control genistein untreated ECFCs). However, the genistein-induced increase in ECFC motile activity was inhibited by ILK siRNA. Next, we examined whether ERK1/2 was involved in genistein-induced proliferation of ECFCs. Genistein increased ERK1/2 activation in ECFC (Figure S1). We found that genistein-stimulated cell proliferation was dependent on ERK1/2 activation in ECFCs. Furthermore, transplanting genistein stimulate-ECFCs (GS-ECFCs) into the ischemic myocardium increased proliferation and survival. However, ERK1/2 inhibitor (U0126, 10^−6^ M)-pretreatment GS-ECFCs showed poor proliferation and survival in ischemic heart tissue. This is the first report to demonstrate the effect of genistein on ECFCs. Our results shed light on the potential role of ERK1/2 in genistein-induced ECFC proliferation.

We next investigated whether genistein enhanced ECFC migration and survival into sites of neovascularization after MI and whether this enhancement leads to greater preservation of myocardial function and integrity. The expression of HGF, SDF-1, and FGF-2 was higher in the genistein stimulate-ECFCs (GS-ECFCs) group compared to the CTRL (control genistein untreated ECFCs) group. We showed *in vivo* that border zone myocardium in the genistein stimulate-ECFCs (GS-ECFCs) group had significantly upregulated levels of HGF, SDF-1, and FGF-2, indicative of an ongoing angiogenic process as late as 4 weeks after ECFC transplantation. Mice injected with genistein stimulate-ECFCs (GS-ECFCs) showed enhanced neovascularization and colocalization of ECFCs to CD31^+^ and α-SMA vascular structures. These ECFCs were either seen in the vicinity of the existing vessels or incorporated into the vascular structures, suggesting the possibility of both engraftment into vascular structures and paracrine mechanisms aiding in neovascularization. These findings, in conjunction with upregulated levels of angiogenic cytokines, are consistent with the known vasculogenic mechanisms of ECFCs. In animals, the preservation or restoration of microvessels is correlated with better LV function after MI, and therapies designed to enhance microvascular circulation preserve cardiac function and integrity [39]. In the present study, the LV function assessment revealed that mice receiving genistein stimulate-ECFCs (GS-ECFCs) showed more improved LV function than those receiving CTRL (control genistein untreated ECFCs). This improved LV function was associated with a reduction in fibrosis size in the genistein stimulate-ECFCs (GS-ECFCs) group.

Ex vivo ECFC priming with genistein before transplantation may thus provide an effective therapeutic strategy to improve the recruitment of injected cells to sites of neovascularization and thereby enhance the efficiency of cell therapy for ischemic vascular disease.

## Materials and Methods

### Ethical statement

After obtaining informed consent, human umbilical cord blood was collected from healthy volunteers according to a protocol approved by the Ethics Review Board of the Hospital of the Pusan National University of YangSan, Korea. We have the list of the volunteers that university hospital can share. The Institutional Animal Care and Use Committee of Pusan National University, YangSan, Korea approved all surgical interventions and post-operative animal care. The consent was written and approved. The approved protocol number is IACUC090017. Therefore, the Ethics Review Board approved this research including both the human and animal studies.

### ECFC culture and stimulation

Human umbilical cord blood (HUCB) was supplied from the Pusan National University Yangsan Hospital (PNUYH, IRB No. 2012-19). ECFCs from human umbilical cord blood (HUCB) were isolated, expanded and characterized as previously described [25]–[26]. The medium was replaced with serum-free EBM-2, 1% FBS for 24 h prior to the experiments. Following various genistein concentrations for incubation 12 h, and used in migration and proliferation assays. When needed, U0126, (ERK1/2 inhibitor, 10^−6^ M) for 30 min prior to genistein treatment, and transfected with ILK, α-parvin, and TRIOBP (F-actin) small interfering RNA (siRNA) (ILK, α-parvin, and TRIOBP-specific siRNA; 200 pmol) for 24 h before genistein treatment. Supernatants and cells were separately after genistein stimulation. All assays were performed in triplicate.

### Animals

Experiments were performed on male 8–10-week-old BALB/CA-nu/nu mice maintained under a 12-h light/dark cycle in accordance with the regulations of Pusan National University. Standard laboratory chow and water were available *ad libitum*. These protocols were approved by the guidelines of the Institutional Animal Care and Use Committee of Pusan National University in Pusan, Korea (IACUC090017).

### Myocardial infarction

Mice were subjected to myocardial infarction (MI) by ligation of left anterior descending coronary artery (LAD) as described previously [2], [3], [27]. Immediately after LAD ligation, one set of mice received intramyocardial injection of ECFCs in a total volume of 15 µL at 5 different sites (basal anterior, mid anterior, mid lateral, apical anterior, and apical lateral) in the periinfarct area. Cytokine secretion, retention, survival, and migration of transplanted ECFCs was assessed after 3 days; LV functional changes on 28 days and structural remodeling at 28 days post-MI.

### In vivo echocardiographic evaluation

An echocardiographic study (Vivid I; GE Healthcare, Piscataway, NJ, USA) using a 11.5 MHz transducer was performed 2 days before and 4 weeks after genistein stimulated-ECFC (GS-ECFC) or CTRL (indicates control genistein untreated ECFC) transplantation. Two-dimensional M-mode traces were obtained at the level of the papillary muscles in at least 3 consecutive cardiac cycles. LV fractional shortening (FS) was calculated as FS  =  (LV end-systolic diameter [LVESD] – LV end-diastolic diameter [LVEDD])/LVEDD (Kawamoto et al., 2001; Kawamoto et al., 2003; Iwasaki et al., 2006).

### Morphometric study

The hearts were perfusion fixed with 10% buffered formalin. Hearts cut into 3 slices (apex, mid-LV and base) and paraffin embedded. The morphometric analysis including infarct size and wall thickness and percent fibrosis was performed on Masson's trichrome stained tissue sections using Image J software (NIH, version 1.30, http://rsb.info.nih.gov/ij/). Wall thickness was measured perpendicular to the infarcted wall at three separate regions and averaged. Fibrosis area and total LV area was measured to determine percent fibrosis.

### TCA precipitation

Filtered culture supernatants were mixed with TCA to a final concentration of 30% (w/v) and were incubated on ice for 30 min or stirred overnight at 4°C. Samples were centrifuged for 20 min at 10,000×g at 4°C. Pellets were washed with ice-cold ethanol (96% [v/v]) and acetone, and were air-dried. Protein pellets were resuspended in 50 mM Tris–HCl (pH 7.5) and incubated for 10 min at 60°C with occasional stirring.

### Western blot analysis

Tissue and cell homogenates (20 μg protein) were separated using 10% sodium dodecyl sulfate-polyacrylamide gel electrophoresis (SDS-PAGE) and transferred to nitrocellulose membranes. After the blots had been washed with TBST (10 mM Tris-HCl [pH 7.6], 150 mM NaCl, 0.05% Tween-20), the membranes were blocked with 5% skim milk for 1 h and incubated with appropriate primary antibodies at the dilutions recommended by the manufacturers. The membranes were then washed, and the primary antibodies were detected using horseradish peroxidase-conjugated goat anti-rabbit IgG or goat anti-mouse IgG secondary antibodies. The bands were visualized by enhanced chemiluminescence (Amersham Biosciences, Buckinghamshire, UK).

### Oris cell migration assay

The Oris Cell Migration Assay and Calcein AM were purchased from Platypus Technologies (Madison, WI, USA) and Invitrogen (Carlsbad, CA, USA), respectively. ECFCs were seeded at 100 µl per well and incubated for 12 h to permit cell adhesion. After cells had reached 70% confluence in the dishes, the inserts were carefully removed and wells were gently washed with culture medium. Then, ECFCs were incubated in fresh medium with or without genistein. ECFC migration was observed microscopically after 12 h. ECFC populations at endpoint were measured by staining with 0.5 mM Calcein AM for 30 min. Migrated ECFCs were quantified by measuring the fluorescence signals using the microplate reader at excitation and emission wavelengths of 485 nm and 515 nm, respectively.

### MTT assay for proliferation

Cell proliferation was determined by MTT [3-(4,5–dimethylthiazol-2-yl)-2,5-diphenyl-tetrazolium bromide] assay. In brief, ECFCs (5×10^3^ cells/well) were plated onto 96-well plates in complete EGM-2 medium. After overnight, cells were serum starved in EBM-2 medium supplemented with 1% FBS for 12 h. They were then cultured in EBM-2 medium supplemented with 1% FBS as control and EBM-2 medium supplemented with 1% FBS and genistein (10^−5^ to 10^−10^ M). After 12 h culture, medium was removed and MTT (5 mg/ml, Sigma) was added to each well. The cells were then incubated at 37°g for 4 hours. The color was extracted with DMSO at 37°7 for 20 min. Relative viable cell numbers were determined by measuring OD_540_ at room temperature.

### Fluorescence-activated cell sorter (FACS) analysis

ECFCs were dissociated in trypsin/EDTA, pelleted by centrifugation and resuspended at ∼10^6^ cells/mL in PBS containing 0.1% BSA. The ECFCs were fixed with 70% ice-cold ethanol for 30 min at 4°C, followed by incubation in a freshly prepared nuclei staining buffer consisting of 250 µg/mL propidium iodide (PI) and 100 µg/mL RNase for 30 min at 37°C. Cell cycle histograms were generated after analyzing the PI-stained cells by FACS (Becton Dickinson). Cell Quest software was used for further analysis.

### Immunohistochemistry

Four weeks after the genistein stimulated-ECFC (GS-ECFC) or CTRL (control untreated ECFC) implantation, mice were euthanized, and their hearts were removed. The excised hearts were retrograde perfused with PBS to wash the coronary vasculature and LV and fixed with 4% paraformaldehyde overnight at 4°C and then with 15% sucrose overnight at 4°C. Each tissue sample was embedded in paraffin or frozen in optimal cutting temperature compound (Tissue-Tek, Sakura, Torrance, CA, USA). Sections (7 µm) stained with hematoxylin and eosin and Masson's trichrome were used to calculate the fibrosis size and the wall thickness by using Image Pro version 4.5 (Media Cybernetics, Bethesda, MD, USA). The sections were subject to immunofluorescent staining with anti-human nuclear antigen (HNA, Chemicon, Temecula, CA, USA) to detect the transplanted human cells. Capillary and arteriole sections of the ischemic regions were subject to immunofluorescent staining with anti-CD31 (Abcam, Cambridge, MA, USA) and anti-smooth muscle α-actin (α-SMA; Abcam), respectively. Primary antibodies against human-specific stromal cell-derived factor-1 (SDF-1; Santa Cruz Biotechnology, Santa Cruz, CA, USA), hepatocyte growth factor (HGF; Santa Cruz Biotechnology), and fibroblast growth factor-2 (FGF-2; BD Transduction Laboratories, Lexington, UK) were used for examining the production of human angiogenic factors in ischemic tissues. Primary antibodies against proliferating cell nuclear antigen (PCNA; Abcam), Ki67 (Abcam), and caspase-3 (Santa Cruz Biotechnology) were used for immunofluorescent staining of ischemic tissues. Sections were counterstained with DAPI (Vector Laboratories, Burlingame, CA, USA) and examined using a FluoView 1000 confocal microscope (Olympus, Tokyo, Japan) with a 600× objective.

### Statistical analyses

Results are presented as mean ± standard error. All experiments were analyzed by analysis of variance (ANOVA). In some experiments, this analysis was followed by a comparison of the treatment means with the control by using the Bonferroni-Dunn test. A *P*-value <0.05 was considered significant. Detailed experimental methods are described in the online-only Data Supplement.

## Supporting Information

Figure S1 Effect of genistein on ERK1/2 activation. ECFCs were treated with genistein for various time periods (0–120 min), and then ERK1/2 phosphorylation was detected by western blotting.
(TIF)

Figure S2 Effect of genistein on estrogen receptor (ER) expression. ECFCs were treated with genistein for 12 h, and ERα and ERβ were detected by western blotting.
(TIF)
