# Supplementary figures and images for: Genistein Promotes Endothelial Colony-Forming Cell (ECFC) Bioactivities and Cardiac Regeneration in Myocardial Infarction
Source: PLoS One. 2014 May 15;9(5):e96155. doi: 10.1371/journal.pone.0096155 (PMC4022670; doi:10.1371/journal.pone.0096155)

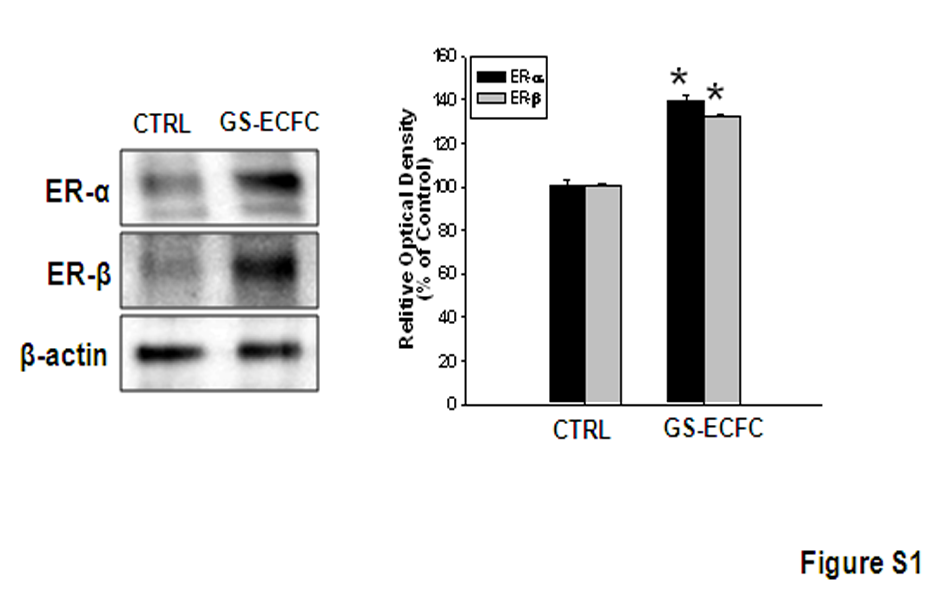

Supplement: Figure S1 — Effect of genistein on ERK1/2 activation. ECFCs were treated with genistein for various time periods (0–120 min), and then ERK1/2 phosphorylation was detected by western blotting. (TIF) [file pone.0096155.s001.tif]

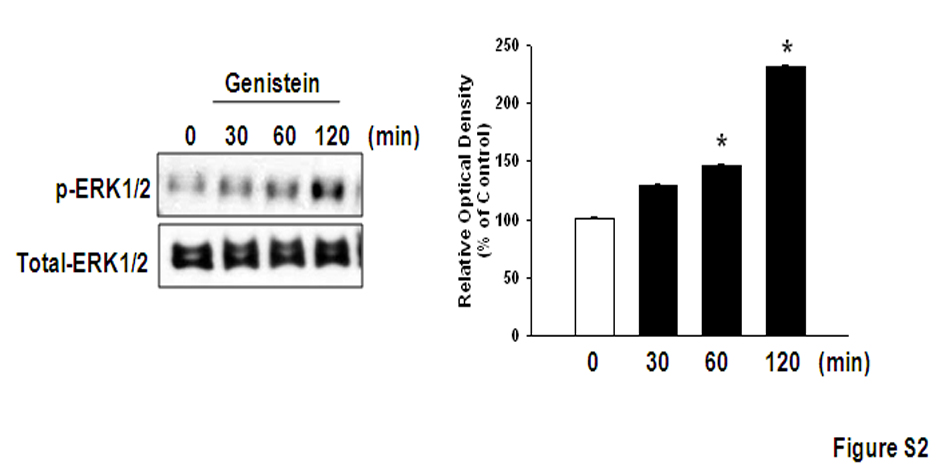

Supplement: Figure S2 — Effect of genistein on estrogen receptor (ER) expression. ECFCs were treated with genistein for 12 h, and ERα and ERβ were detected by western blotting. (TIF) [file pone.0096155.s002.tif]
